# Supplementary material for: Consumption of Energy Drinks and Attitudes Among School Students Following the Ban on Sales to Minors in Poland
Source: Nutrients. 2025 Oct 8;17(19):3167. doi: 10.3390/nu17193167 (PMC12526465; doi:10.3390/nu17193167)
Supplement: Supplementary file 1 [file nutrients-17-03167-s001.zip › nutrients-3884851-supplementary.pdf]

Supplementary File S1.

ANONYMOUS QUESTIONNAIRE

Age completed (years): …… Primary school student ☐ Secondary school student ☐

Gender: female ☐ male ☐ Place of residence: urban area ☐ rural area ☐

1. Have you heard about the introduction of a ban on the sale of energy drinks to individuals under 18 years of age in Poland?

   ☐ Yes

   ☐ No

2. Have you tried buying energy drinks in a physical shops in the last year?

   ☐ Yes

   ☐ No

3. If you have tried to buy such drinks, did the seller ask for proof of age? (only to be filled out by those who answered „Yes” in question no. 2)

   ☐ Always

   ☐ Sometimes

   ☐ Never

4. In case of an attempt to purchase, did the seller refuse to sell you the drinks? (only to be filled out by those who answered "Yes" in question no. 2)

   ☐ Always

   ☐ Sometimes

   ☐ Never

5. Have you tried to buy energy drinks online/shopping app in the last year?

   ☐ Yes

   ☐ No

6. If you tried to buy such drinks online/shopping app, were you able to make the purchase on your own? (only to be filled out by those who answered „Yes” in question no. 5)

   ☐ Always

   ☐ Sometimes

☐ Never

7. Is there a belief among your peers that sellers always comply with the ban on selling energy drinks to minors?

☐ Yes

☐ No

8. Do you generally think that the ban on selling energy drinks means that obtaining such products by minors:

☐ It is not possible

☐ Does not pose a big problem, because (provide an explanation)…………………

……………………………………………………………………………………………

……………………………………………………………………………………………

……………………………………………………………………………………………

9. Have you consumed energy drinks in the last month?

☐ Yes — choose one answer →

☐ Everyday or almost everyday

☐ 3-4 times per week

☐ 1-2 times per week

☐ 1-2 times per month

☐ No

10. Why do you drink energy drinks? You can select multiple answers (only to be filled out by those who answered „Yes" in question no. 9)

☐ To increase physical and mental performance

☐ To avoid feeling excluded from the peer group

☐ Because they are tasty

☐ Because they are „fashionable"

☐ Another reason (provide an explanation) ……………………………………………

……………………………………………………………………………………...

11. Has the establishment of a legal ban on the sale of energy drinks to individuals under 18 years of age convinced you that they have a harmful effect on the health of children and adolescents?

☐ Yes

☐ No
